# Restoration by the Use of Silicate Cements

**Published:** 1912-11-15

**Authors:** C. O. Rhea

**Affiliations:** Nashville, Tenn.


### RESTORATION BY THE USE OF SILICATE CEMENTS

BY C. O. RHEA, D. D. S., NASHVILLE, TENN.

Read before Tennessee State Dental Society, Juny 6, 1912.

We live in the age of invention. The success of yesterday is not sufficient for today. Arts and Crafts, Science and Invention, are making rapid progress to a goal known as "Perfection," which the human mind can only conceive.

That branch of science known as Dental Surgery, we are proud to claim, is not lagging behind in its progress to this goal.

No longer does our profession tolerate the unsightly gold crowns and massive gold restorations, which could only do credit to the Jeweler's art, and the strong appeal to the aesthetic has stirred the heart and ambition of every true follower of his profession. The task of educating the public to the standard of perfection to which we have attained in this line has made itself known in almost every locality.

There was a time when gold crowns on centrals, laterals, and cuspids, were considered elegant; and at that time they were, for it was the height to which the art of restoration had progressed. That era has passed, and we find that even among the most illiterate an anterior shell crown is repulsive.

The term beautiful, as applied to gold crowns and fillings, does not well conform to our standard of taste. They may be beautifully constructed, but their lack of harmony in color presents a glowing evidence of decay and repair.

Such public display of the deteriorations of our bodies would be grossly shocking to our refined natures, were they not necessary.

The time is rapidly approaching when the necessity for such things will be no more, and their *beauty* will be turned to shocking conspicuousness. Just as the anterior shell crown within the past generation has passed almost into oblivion just so will the conspicuous anterior gold fillings pass within the present generation, and give place to the more aesthetic means of restoration which we now have in the use of Silicate Cements.

There are on the market today several similar cement preparations, put out by different manufacturers and under different names, but all classified under the term, "Silicate Cement." They all possess similar properties and aim exclusively at durability, strength, simplicity, and harmony—the four words which characterize our modern ideas in art, dress, and speech.

As to which of these several preparations one should use, is for the individual operator himself to decide. I have found from extensive investigation that one brand may be considered a success in the hands of one operator, and may be a failure in the hands of another. They are all sensitive materials, and the art of individual manipulation must necessarily be accomplished before assured success will ensue.

Five years ago I began the use of Silicate Cements. Being a beginner in the profession, and using a material which was just making its advent into the restoration of the dental organs, my work was undertaken with a high degree of anxiety, and executed with the utmost precaution.

I am proud to state that some of those first efforts are today doing credit to the art of aesthetic restoration. Two years since, I read before the Tennessee State Dental Association a paper on "Artificial Enamel," in which I set forth my experience with this material and gave such practical hints concerning its use and method of manipulation, as I had learned from the literature and my own practical experience. It is sufficient for me to say today that I still adhere to these general principles, and have no reason to change my method or to withdraw any claim I made for this particular brand of Silicate Cement.

Gentlemen, do not understand me to say that all my Silicate Cement fillings stand, or that they are all perfectly matched in shade and shape, for to this standard of perfection

I have not yet attained. Neither do all of my gold or silver fillings indefinitely withstand the ravages of decay, nor boldly defy the perilous laws of disintegration.

Do not make the mistake of thinking the Silicate Cement filling material wholly ideal, or that it can be honestly claimed for it that it will do what gold will in resisting the destructive forces of the oral cavity; for, while it has some advantages over gold, it is lacking in some very important features, such as *edge strength* and *resistance.* Silicate Cement fillings should be inserted only when indicated as they have their limitations; and it is for the conservative operator, not only to master the art of manipulation, but to be able to determine where it is definitely indicated, and to produce the correct cavity formation.

The deficiency in edge strength limits its use in occlusal and incisial cavities, yet these difficulties can be overcome by one of the most effective means of restoration, the use of the gold inlay, inlaid with a Silicate Cement filling on the labial surface.

The perfect filling material has not yet been found; but according to the physical properties of the material, combined with the degree of skill used in the manipulation, does our work approach the standard of perfection—and it is my conviction that the Silicate Cements have their well-deserved place high in the scale of restorative materials.

We know good only by comparison with bad—
We measure success with failure;
We appreciate the beautiful because the ugly exists;
We strive at the aesthetic, and nature resists.
However the strife is worth the while,
So we toil untiringly for perfection;
And our efforts are rewarded with success
When we are sure we have done our very best.

